# Supplementary material for: Molecular Alterations in TP53, WNT, PI3K, TGF-Beta, and RTK/RAS Pathways in Gastric Cancer Among Ethnically Heterogeneous Cohorts
Source: Cancers (Basel). 2025 Mar 23;17(7):1075. doi: 10.3390/cancers17071075 (PMC11987813; doi:10.3390/cancers17071075)
Supplement: Supplementary file 1 [file cancers-17-01075-s001.zip › cancers-3519563-supplementary.pdf]

Supplementary Materials:

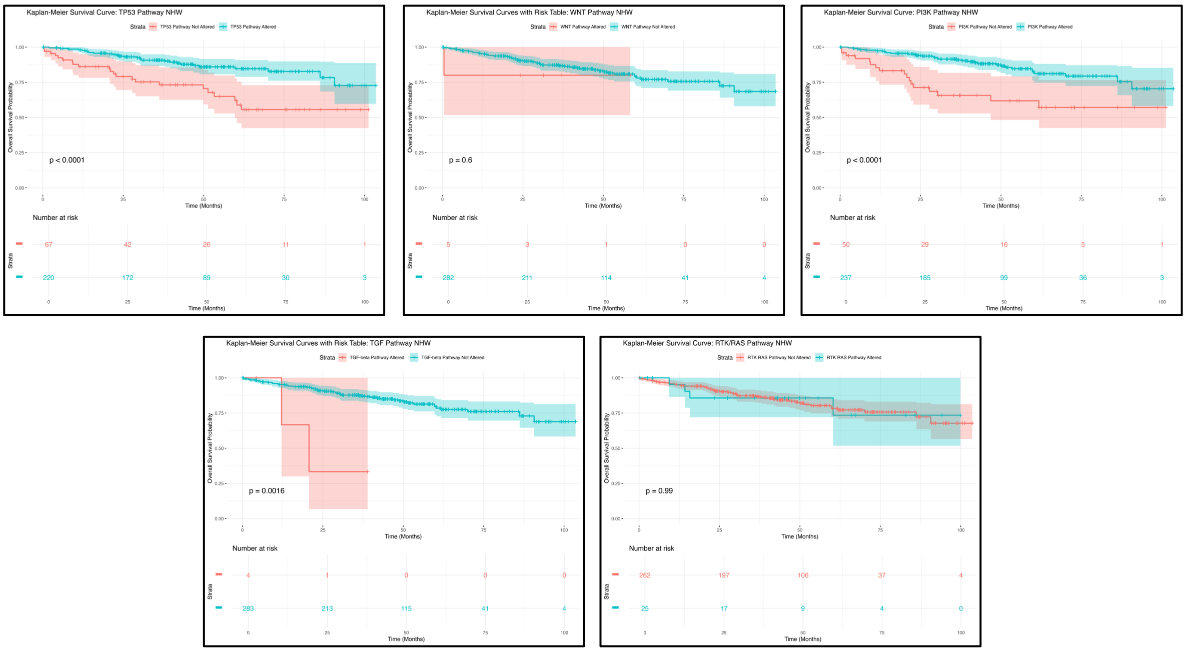

Figure S1. Kaplan-Meier overall survival curves for Non-Hispanic White (NHW) gastric cancer (GC) patients, stratified by the presence or absence of TP53 (upper left), WNT (upper middle), PI3K (upper right), TGF-Beta (lower left), and RTK/RAS (lower right) pathway alterations.

Table S1. Alteration rates of TP53, WNT, PI3K, TGF-Beta and RTK/RAS pathway-related genes among Hispanic/Latino (H/L) and Non-Hispanic White (NHW) gastric cancer (GC) patients.

TP53 Pathway			
**Gene**	**H/L Samples n (%)**	**NHW Samples n (%)**	**p-value**
**TP53 Mutation**			
Present	5 (6.0%)	32 (4.5%)	0.5765
Absent	78 (94.0%)	685 (95.5%)	
**MDM2 Mutation**			
Present	0 (0.0%)	0 (0.0%)	1
Absent	83 (100.0%)	717 (100.0%)	
**MDM4 Mutation**			
Present	0 (0.0%)	4 (0.6%)	1
Absent	83 (100.0%)	713 (99.4%)	
**CDKN2A Mutation**			
Present	1 (1.2%)	4 (0.6%)	0.4225
Absent	82 (98.8%)	713 (99.4%)	
**ATM Mutation**			
Present	1 (1.2%)	19 (2.6%)	0.7118
Absent	82 (98.8%)	698 (97.4%)	
**CHEK2 Mutation**			
Present	0 (0.0%)	5 (0.7%)	1
Absent	83 (100.0%)	712 (99.3%)	
**RPS6KA3 Mutation**			
Present	0 (0.0%)	0 (0.0%)	1

Absent	83 (100.0%)	717 (100.0%)	
**WNT Pathway**			
**Gene**	**H/L Samples n (%)**	**NHW Samples n (%)**	**p-value**
**AMER1 Mutation**			
Present	0 (0.0%)	0 (0.0%)	1
Absent	83 (100.0%)	717 (100.0%)	
**APC Mutation**			
Present	3 (3.6%)	6 (0.8%)	0.05708
Absent	80 (96.4%)	711 (99.2%)	
**AXIN1 Mutation**			
Present	0 (0.0%)	0 (0.0%)	1
Absent	83 (100.0%)	717 (100.0%)	
**AXIN2 Mutation**			
Present	0 (0.0%)	2 (0.3%)	1
Absent	83 (100.0%)	715 (99.7%)	
**CTNNB1 Mutation**			
Present	1 (1.2%)	3 (0.4%)	0.3553
Absent	82 (98.8%)	714 (99.6%)	
**DKK1 Mutation**			
Present	0 (0.0%)	0 (0.0%)	1
Absent	83 (100.0%)	717 (100.0%)	
**DKK2 Mutation**			
Present	0 (0.0%)	0 (0.0%)	1
Absent	83 (100.0%)	717 (100.0%)	
**DKK3 Mutation**			
Present	0 (0.0%)	0 (0.0%)	1
Absent	83 (100.0%)	717 (100.0%)	
**DKK4 Mutation**			
Present	0 (0.0%)	0 (0.0%)	1
Absent	83 (100.0%)	717 (100.0%)	
**GSK3B Mutation**			
Present	0 (0.0%)	0 (0.0%)	1
Absent	83 (100.0%)	717 (100.0%)	
**LRP5 Mutation**			
Present	0 (0.0%)	0 (0.0%)	1
Absent	83 (100.0%)	717 (100.0%)	
**LRP6 Mutation**			
Present	0 (0.0%)	0 (0.0%)	1
Absent	83 (100.0%)	717 (100.0%)	
**RNF43 Mutation**			
Present	0 (0.0%)	2 (0.3%)	1
Absent	83 (100.0%)	715 (99.7%)	
**SFRP1 Mutation**			
Present	0 (0.0%)	0 (0.0%)	1
Absent	83 (100.0%)	717 (100.0%)	
**SFRP2 Mutation**			
Present	0 (0.0%)	0 (0.0%)	1
Absent	83 (100.0%)	717 (100.0%)	
**SFRP3 Mutation**			
Present	0 (0.0%)	0 (0.0%)	1
Absent	83 (100.0%)	717 (100.0%)	
**SFRP4 Mutation**			
Present	0 (0.0%)	0 (0.0%)	1

Absent	83 (100.0%)	717 (100.0%)	
**SFRP5 Mutation**			
Present	0 (0.0%)	0 (0.0%)	1
Absent	83 (100.0%)	717 (100.0%)	
**TCF7 Mutation**			
Present	0 (0.0%)	0 (0.0%)	1
Absent	83 (100.0%)	717 (100.0%)	
**TCF7L1 Mutation**			
Present	1 (1.2%)	1 (0.1%)	0.1969
Absent	82 (98.8%)	716 (99.9%)	
**TCF7L2 Mutation**			
Present	1 (1.2%)	4 (0.6%)	0.4225
Absent	82 (98.8%)	713 (99.4%)	
**TLE1 Mutation**			
Present	0 (0.0%)	0 (0.0%)	1
Absent	83 (100.0%)	717 (100.0%)	
**TLE2 Mutation**			
Present	0 (0.0%)	0 (0.0%)	1
Absent	83 (100.0%)	717 (100.0%)	
**TLE3 Mutation**			
Present	0 (0.0%)	0 (0.0%)	1
Absent	83 (100.0%)	717 (100.0%)	
**TLE4 Mutation**			
Present	0 (0.0%)	0 (0.0%)	1
Absent	83 (100.0%)	717 (100.0%)	
**WIF1 Mutation**			
Present	0 (0.0%)	0 (0.0%)	1
Absent	83 (100.0%)	717 (100.0%)	
**PI3K Pathway**			
**Gene**	**H/L Samples n (%)**	**NHW Samples n (%)**	**p-value**
**PTEN Mutation**			
Present	3 (3.6%)	13 (1.8%)	0.2257
Absent	80 (96.4%)	704 (98.2%)	
**PIK3R1 Mutation**			
Present	0 (0.0%)	7 (1.0%)	1
Absent	83 (100.0%)	710 (99.0%)	
**PIK3R2 Mutation**			
Present	0 (0.0%)	1 (0.1%)	1
Absent	83 (100.0%)	716 (99.9%)	
**PIK3R3 Mutation**			
Present	0 (0.0%)	0 (0.0%)	1
Absent	83 (100.0%)	717 (100.0%)	
**PIK3CA Mutation**			
Present	3 (3.6%)	13 (1.8%)	0.2257
Absent	80 (96.4%)	704 (98.2%)	
**INPP4B Mutation**			
Present	0 (0.0%)	0 (0.0%)	1
Absent	83 (100.0%)	717 (100.0%)	
**AKT1 Mutation**			
Present	0 (0.0%)	2 (0.3%)	1
Absent	83 (100.0%)	715 (99.7%)	
**AKT2 Mutation**			
Present	0 (0.0%)	4 (0.6%)	1

Absent	83 (100.0%)	713 (99.4%)	
**AKT3 Mutation**			
Present	0 (0.0%)	0 (0.0%)	1
Absent	83 (100.0%)	717 (100.0%)	
**PPP2R1A Mutation**			
Present	0 (0.0%)	1 (0.1%)	1
Absent	83 (100.0%)	716 (99.9%)	
**TSC1 Mutation**			
Present	0 (0.0%)	12 (1.7%)	0.6247
Absent	83 (100.0%)	705 (98.3%)	
**TSC2 Mutation**			
Present	1 (1.2%)	13 (1.8%)	1
Absent	82 (98.8%)	704 (98.2%)	
**STK11 Mutation**			
Present	2 (2.4%)	7 (1.0%)	0.2377
Absent	81 (97.6%)	710 (99.0%)	
**RHEB Mutation**			
Present	0 (0.0%)	0 (0.0%)	1
Absent	83 (100.0%)	717 (100.0%)	
**RICTOR Mutation**			
Present	0 (0.0%)	3 (0.4%)	1
Absent	83 (100.0%)	714 (99.6%)	
**MTOR Mutation**			
Present	0 (0.0%)	11 (1.5%)	0.616
Absent	83 (100.0%)	706 (98.5%)	
**RPTOR Mutation**			
Present	1 (1.2%)	3 (0.4%)	0.3553
Absent	82 (98.8%)	714 (99.6%)	
**TGF-beta Pathway**			
**Gene**	**H/L Samples n (%)**	**NHW Samples n (%)**	**p-value**
**ACVR2A Mutation**			
Present	0 (0.0%)	0 (0.0%)	1
Absent	83 (100.0%)	717 (100.0%)	
**ACVR2B Mutation**			
Present	0 (0.0%)	0 (0.0%)	1
Absent	83 (100.0%)	717 (100.0%)	
**SMAD2 Mutation**			
Present	0 (0.0%)	2 (0.3%)	1
Absent	83 (100.0%)	715 (99.7%)	
**SMAD3 Mutation**			
Present	0 (0.0%)	0 (0.0%)	1
Absent	83 (100.0%)	717 (100.0%)	
**SMAD4 Mutation**			
Present	2 (2.4%)	3 (0.4%)	0.08645
Absent	81 (97.6%)	714 (99.6%)	
**TGFBR1 Mutation**			
Present	0 (0.0%)	0 (0.0%)	1
Absent	83 (100.0%)	717 (100.0%)	
**TGFBR2 Mutation**			
Present	0 (0.0%)	2 (0.3%)	1
Absent	83 (100.0%)	715 (99.7%)	
**RTK/RAS Pathway**			

Gene	H/L Samples n (%)	NHW Samples n (%)	p-value
**EGFR Mutation**			
Present	3 (3.6%)	7 (1.0%)	0.07558
Absent	80 (96.4%)	710 (99.0%)	
**ERBB2 Mutation**			
Present	0 (0.0%)	2 (0.3%)	1
Absent	83 (100.0%)	715 (99.7%)	
**ERBB4 Mutation**			
Present	2 (2.4%)	9 (1.3%)	0.3187
Absent	81 (97.6%)	708 (98.7%)	
**MET Mutation**			
Present	1 (1.2%)	7 (1.0%)	0.5859
Absent	82 (98.8%)	709 (98.9%)	
**PDGFRA Mutation**			
Present	10 (12.0%)	93 (13.0%)	1
Absent	73 (88.0%)	624 (87.0%)	
**FGFR1 Mutation**			
Present	3 (3.6%)	6 (0.8%)	0.05708
Absent	80 (96.4%)	711 (99.2%)	
**FGFR2 Mutation**			
Present	2 (2.4%)	2 (0.3%)	0.05557
Absent	81 (97.6%)	715 (99.7%)	
**FGFR3 Mutation**			
Present	2 (2.4%)	5 (0.7%)	0.1585
Absent	81 (97.6%)	712 (99.3%)	
**FGFR4 Mutation**			
Present	0 (0.0%)	4 (0.6%)	1
Absent	83 (100.0%)	713 (99.4%)	
**KIT Mutation**			
Present	59 (71.1%)	503 (70.2%)	0.8998
Absent	24 (28.9%)	214 (29.8%)	
**IGF1R Mutation**			
Present	0 (0.0%)	0 (0.0%)	1
Absent	83 (100.0%)	717 (100.0%)	
**RET Mutation**			
Present	0 (0.0%)	6 (0.8%)	1
Absent	83 (100.0%)	711 (99.2%)	
**ROS1 Mutation**			
Present	0 (0.0%)	13 (1.8%)	0.3813
Absent	83 (100.0%)	704 (98.2%)	
**ALK Mutation**			
Present	0 (0.0%)	5 (0.7%)	1
Absent	83 (100.0%)	712 (99.3%)	
**FLT3 Mutation**			
Present	1 (1.2%)	4 (0.6%)	0.4225
Absent	82 (98.8%)	713 (99.4%)	
**NTRK1 Mutation**			
Present	0 (0.0%)	4 (0.6%)	1
Absent	83 (100.0%)	713 (99.4%)	
**NTRK2 Mutation**			
Present	0 (0.0%)	1 (0.1%)	1
Absent	83 (100.0%)	716 (99.9%)	
**CBL Mutation**			

Present	0 (0.0%)	6 (0.8%)	1
Absent	83 (100.0%)	711 (99.2%)	
**ERRFI1 Mutation**			
Present	0 (0.0%)	0 (0.0%)	1
Absent	83 (100.0%)	717 (100.0%)	
**SOS1 Mutation**			
Present	0 (0.0%)	2 (0.3%)	1
Absent	83 (100.0%)	715 (99.7%)	
**NF1 Mutation**			
Present	3 (3.6%)	32 (4.5%)	1
Absent	80 (96.4%)	685 (95.5%)	
**RASA1 Mutation**			
Present	0 (0.0%)	3 (0.4%)	1
Absent	83 (100.0%)	714 (99.6%)	
**PTPN11 Mutation**			
Present	2 (2.4%)	2 (0.3%)	0.05557
Absent	81 (97.6%)	715 (99.7%)	
**KRAS Mutation**			
Present	1 (1.2%)	5 (0.7%)	0.4828
Absent	82 (98.8%)	712 (99.3%)	
**HRAS Mutation**			
Present	0 (0.0%)	2 (0.3%)	1
Absent	83 (100.0%)	715 (99.7%)	
**NRAS Mutation**			
Present	1 (1.2%)	2 (0.3%)	0.2804
Absent	82 (98.8%)	715 (99.7%)	
**RIT1 Mutation**			
Present	0 (0.0%)	1 (0.1%)	1
Absent	83 (100.0%)	716 (99.9%)	
**ARAF Mutation**			
Present	0 (0.0%)	2 (0.3%)	1
Absent	83 (100.0%)	715 (99.7%)	
**BRAF Mutation**			
Present	1 (1.2%)	6 (0.8%)	0.5369
Absent	82 (98.8%)	711 (99.2%)	
**RAF1 Mutation**			
Present	0 (0.0%)	4 (0.6%)	1
Absent	83 (100.0%)	713 (99.4%)	
**RAC1 Mutation**			
Present	0 (0.0%)	0 (0.0%)	1
Absent	83 (100.0%)	717 (100.0%)	
**MAPK1 Mutation**			
Present	0 (0.0%)	0 (0.0%)	1
Absent	83 (100.0%)	717 (100.0%)	
**MAP2K1 Mutation**			
Present	0 (0.0%)	0 (0.0%)	1
Absent	83 (100.0%)	717 (100.0%)	
**MAP2K2 Mutation**			
Present	0 (0.0%)	2 (0.3%)	1
Absent	83 (100.0%)	715 (99.7%)	
